# The use of procalcitonin in the determination of severity of sepsis, patient outcomes and infection characteristics

**DOI:** 10.1371/journal.pone.0206527

**Published:** 2018-11-14

**Authors:** Iram Yunus, Anum Fasih, Yanzhi Wang

**Affiliations:** 1 Department of Family Medicine, The University of Illinois, College of Medicine at Peoria, Peoria, IL, United States of America; 2 Department of Medicine, University of Illinois, College of Medicine at Peoria, Peoria, IL, United States of America; Azienda Ospedaliero Universitaria Careggi, ITALY

## Abstract

**Objective:**

The primary objective of this study was to determine the correlation between procalcitonin values and illness severity by evaluating the degree of end organ dysfunction using the Sequential Organ Failure Assessment score, length of stay and the severity of sepsis (sepsis alone vs. septic shock), The hypothesis that procalcitonin values would be higher in sicker patients was formulated before data collection began. Secondary outcomes studied in relation to procalcitonin levels included infection characteristics such as the site of infection, microbial agent and dialysis dependent CKD.

**Design:**

Unblinded retrospective cohort study. September 2014-December 2016.

**Setting:**

364 patients with a diagnosis of sepsis or severe sepsis who were admitted to the general medical ward and ICU at Methodist Medical Center and Proctor Hospital in Peoria, Illinois, USA.

**Results:**

This study demonstrates the following: Weak positive correlation between procalcitonin and SOFA score. Negligible correlation with length of stay. Higher values in patients who died than in patients who survived to discharge (p = 0.058). Sensitivity and specificity of procalcitonin for septic shock was 63 and 65% respectively. Sites typically infected by gram negative bacteria have higher procalcitonin values than sites infected by gram positive bacteria (p = 0.03). Higher procalcitonin in bacteremia than non-bacteremic infections (p = 0.004). Higher procalcitonin in dialysis-dependent CKD patients (p = 0.020).

**Conclusions:**

Procalcitonin has a higher specificity for bacterial infections than other acute phase reactants. Although initial procalcitonin value may be helpful in the determination of illness severity, it is not always a reliable prognostic indicator and carries little significance as a standalone value. Procalcitonin values may be influenced by preexisting comorbid conditions such as chronic kidney disease, which are associated with higher procalcitonin values at baseline. Procalcitonin can provide invaluable information when viewed as one piece of a clinical puzzle, and is most powerful when the interpreting physician is aware of how values are influenced by the different clinical scenarios presented in this article.

## Introduction

Sepsis is a clinical syndrome with high mortality rates demonstrating a directly proportionate relationship to disease severity [1]. Therefore, the study of biomarkers for sepsis has been area of interest and research. The most important feature of a biomarker is its potential to influence clinical decision-making. Procalcitonin is one such marker that has shown great promise in identifying sepsis, assessing severity of illness and guiding antibiotic management[2, 3, 4].

Procalcitonin is a peptide precursor of calcitonin and is part of the inflammatory cascade in sepsis. Procalcitonin levels tend to be elevated in bacterial infections whereas they are depressed in viral infections [4], and high PCT has been known to predict bacteremia [5]. Procalcitonin is detectable in the serum within 4 hours and has a half-life of 22–26 hours [6]. Peak levels occur between 12 and 48 hours [7, 8]. However, PCT levels may be elevated in patients who do not have sepsis, with levels between 2–10 ng/mL seen in patients with conditions such as autoimmune disorders [9], trauma [10], cardiac arrest [11], surgery [12], burns [13] and pancreatitis [14].

However, a 2012 systematic review and meta-analysis of nine observational studies that evaluated procalcitonin as a marker of infection in patients with autoimmune disease found that procalcitonin and CRP exhibited similar sensitivity for infection (75 versus 77 percent), but that procalcitonin had significantly higher specificity (90 versus 56 percent) [15].One large multicenter observational study of procalcitonin levels in ICU patients with pneumonia requiring mechanical ventilation found that PCT levels were higher in non-survivors than in survivors, and that initial and maximum PCT levels correlated with maximum Sequential Organ Failure Assessment (SOFA) score [3].In one small study, procalcitonin levels were shown to increase over time in non-survivors of sepsis and decrease in survivors [16].

However, there is conflicting data about the utility of procalcitonin when used as a guide to implementing appropriate antibiotic usage and as a predictor of patient outcomes. One large study found that even though patients with positive blood cultures received antibiotics earlier when they were monitored with procalcitonin, the use of PCT algorithms for the escalation of antibiotic treatment was detrimental to end-organ health and resulted in increased length of ICU stay[17].In contrast to this, a smaller study which measured the duration of antibiotic therapy using a PCT based algorithm found that duration of therapy was less in the PCT guided patient group (9.5 days) in comparison to the control group (13 days)[18].

The primary objective of this study was to determine the correlation between initial procalcitonin values and mortality, illness severity and prognosis by evaluating the degree of end organ dysfunction using the Sequential Organ Failure Assessment (SOFA) score, length of stay and the severity of sepsis (sepsis alone vs septic shock). The hypothesis that procalcitonin values would be higher in sicker patients was formulated before data collection began. A secondary objective was to evaluate procalcitonin association with infection characteristics such as site of infection, extent of infection, and etiological agent (gram positive bacteria, gram negative bacteria versus viral infection) and dialysis dependent CKD. This study also attempted to determine a procalcitonin threshold for septic shock.

## Methods

This was a retrospective chart review that included male and female patients aged 20–79 who were admitted to the Methodist Medical Center and Proctor Hospital between September 2014 and December 2016. This study was approved by the Institutional Review Board of the University of Illinois, College of Medicine at Peoria. Based on ICD-9 and 10, patient charts containing procalcitonin measurements and carrying the diagnoses of sepsis, severe sepsis and septic shock were retrieved for data analysis. In keeping with the recent Surviving Sepsis guidelines [19]in which severe sepsis is no longer considered a distinct category, patients with a diagnosis of severe sepsis were included in the ‘sepsis’ group. Septic shock is defined as a condition that requires the use of vasopressors in order to maintain a MAP of 65 mmHg or above and a persistent lactate of >2 mmol/L in spite of adequate fluid resuscitation [20].Table 1 outlines patient demographics, mean and median initial procalcitonin values and the frequency of occurrence of comorbid conditions in the given patient population.

**Table 1:** Patient demographics, mean and median procalcitonin values, frequency of occurrence of comorbid conditions.

Variable		N (%)
Age	Mean ± SD	61.3 ± 12.6
	Median (min-max)	63.0 (20.0–86.0)
Sex	Male	189 (51.9)
	Female	175 (48.1)
Initial PCT value	Mean ± SD	13.9 ± 31.6
	Median (min-max)	1.6 (0.1–252.5)
Diabetes mellitus		135 (37.1)
Hypertension		212 (58.2)
COPD		109 (29.9)
Coronary artery disease		81 (22.3)
Congestive heart failure		72 (19.8)
CKD without dialysis		51 (14.0)
CKD with dialysis		16 (4.4)
Atrial fibrillation		49 (13.5)
Pulmonary embolism		6 (1.6)
Cancer		71 (19.5)

Given that procalcitonin elevations may be seen in conditions other than sepsis, patients who had undergone major surgery in the 30 days prior to presentation (n = 19), those who had sustained severe trauma (n = 0) or major burns (n = 0), noncompliant dialysis dependent patients (n = 5), and patients with medullary thyroid carcinoma (n = 0) were excluded from this study. Thus, only patients with confirmed infection were studied, and patients who did not have radiologic or microbiologic proof of infection as seen imaging or culture (n = 84) were excluded from the study. Starting sample size was 488 of which 124 patients were excluded, with a final cohort numbering 364.

Sequential organ function assessment (SOFA) scores and length of stay for each patient were determined. Where an arterial blood gas was unavailable, partial pressure of oxygen (PaO_2_) was approximated using the standard oxygen-hemoglobin dissociation curve by determining the oxygen saturation on pulse oximetry and plotting this value against PaO2. Patient outcome was determined based on four pre-defined possibilities. These were as follows:

1. Patient improves and is discharged;
2. Patient dies on the ward floor;
3. Patient is admitted to/transferred to the ICU and dies;
4. Patient is admitted to/transferred to the ICU, improves and is subsequently discharged.

Data regarding comorbidities was also collected. These included diabetes mellitus, hypertension, coronary artery disease, atrial fibrillation, chronic kidney disease (CKD), as defined by the ICD-9 coding system, congestive heart failure, pulmonary embolism and cancer. Of these, data analysis was limited to CKD, which has been associated with higher baseline procalcitonin values [21, 22]. Additionally, infection characteristics were evaluated, including anatomical site (pulmonary, genitourinary, gastrointestinal and musculoskeletal [including cellulitis, osteomyelitis and septic arthritis]), extent of infection (non-bacteremia vs. bacteremia), and etiological agent (gram-positive bacteria, gram-negative bacteria vs. viral).

Spearman correlation coefficients were determined for procalcitonin and organ dysfunction (as determined by the SOFA score), procalcitonin and length of stay, and procalcitonin and age. The Kruskal-Wallis test was used to assess the association between procalcitonin values and outcome, anatomical site of infection and the etiological microorganism. The Wilcoxon rank-sum test was used to evaluate the association between procalcitonin and dialysis-dependent CKD, procalcitonin and sepsis versus septic shock, and procalcitonin and extent of infection. The hypothesis that procalcitonin values would be higher in sicker patients was formulated before data collection began.

## Results

### Organ dysfunction (SOFA score)

These results demonstrated a low positive correlation between PCT and SOFA score (0.33, p-value <0.001) indicating that as PCT values rose, end organ dysfunction worsened. The correlation between PCT and length of stay (LOS) was negligible as was that between PCT and age. This suggests that initial PCT values are not predictive of length of stay and that there is no relationship between PCT and age. Table 2A lists mean PCT values for SOFA score, length of stay and age. Table 3 outlines relates the numerical size of the correlation to the interpretation of the result.

**Table 2:** Correlation between procalcitonin (PCT), SOFA score, Length of Stay (LOS) and age.

Variable	Initial PCT value
SOFA score	0.3293
LOS	0.1721
Age	0.0133

**Table 3:** Relationship between numerical size of the correlation and the interpretation.

Size of correlation	Interpretation
0.90 to 1.00 (-0.90 to -1.00)	Very high positive (negative) correlation
0.70 to 0.90 (-0.70 to -0.90)	High positive (negative) correlation
0.50 to 0.70 (-0.50 to– 0.70)	Moderate positive (negative) correlation
0.30 to 0.50 (-0.30 to -0.50)	Low positive (negative) correlation
0.00 to 0.30 (0.00 to -0.30)	Negligible correlation

### Outcome

PCT values were compared among four outcomes using the Kruskal-Wallis test. At a significance level of 0.05, PCT values are not statistically significantly different among four outcomes (p value = 0.058). The mean procalcitonin value did not seem to have a strong correlation with clinical outcome (Table 4), with initial values being only marginally higher in patients who died during admission in comparison to those that survived to discharge. Mean PCT values were 7 ± 17 and 17.4 ± 36.8 in patients that were admitted to the floor or to the ICU respectively and subsequently discharged. Mean PCT values were slightly higher in patients who died on the floor or in the ICU (21.7 ± 36 and 19.3 ± 38.3 respectively. p = 0.058).

**Table 4:** Correlation between PCT value and outcome.

Variables	Total N = 364	A N = 133	B N = 3	C N = 45	D N = 183
Median PCT	1.6 (0.1–252.5)	1.5 (0.1–131.0)	1.7 (0.2–63.2)	2.0 (0.1–173.1)	1.8 (0.1–252.5)
Mean PCT ± SD	13.9 ± 31.6	7.0 ± 17.0	21.7 ± 36.0	19.3 ± 38.3	17.4 ± 36.8

A = Patient improves and is discharged.

B = Patient dies on the ward floor.

C = Patient is admitted to or transferred to the ICU and dies.

D = Patient is admitted to or transferred to the ICU and is subsequently discharged.

### Extent of infection

PCT values were compared between bacteremia group and non-bacteremic infection group using Wilcoxon rank-sum test. At significance level of 0.05, PCT values are statistically significantly different between two groups, p value = 0.004.

Mean PCT was found to be higher in patients with bacteremia as demonstrated by positive blood cultures (18.3±33.7) in comparison to patients with non-bacteremic infection and negative blood cultures (12.8 ± 31) [P = 0.04]. These findings are demonstrated in Table 5.

**Table 5:** Correlation between PCT and extent of infection.

Variables	Total N = 363	Bacteremia N = 73	Non-bacteremic infection N = 290
Median PCT	1.7 (0.1–252.5)	4.7 (0.1–173.1)	1.4 (0.1–252.5)
Mean PCT ± SD	13.9 ± 31.6	18.3 ± 33.7	12.8 ± 31.0

### Sepsis severity (Sepsis vs septic shock)

Procalcitonin values demonstrated a statistically significant and directly proportionate relationship to severity of sepsis as determined by septic shock necessitating vasopressors (Table 6). Mean procalcitonin was 32.7 ± 52.2 in patients with septic shock requiring vasopressors and 9.6 ± 22.7 in patients with sepsis alone (p< = 0.01). At a value of 3.05 ng/mL, the sensitivity and specificity of PCT for septic shock was found to be 63% and 65% respectively.

**Table 6:** Correlation between PCT and severity of sepsis.

Variables	Total N = 364	Shock N = 67	Sepsis N = 297
Median PCT	1.6 (0.1–252.5)	8.1 (0.1–252.5)	1.4 (0.1–200.0)
Mean PCT ± SD	13.9 ± 31.6	32.7 ± 52.2	9.6 ± 22.7

### Microorganism

The association between PCT and infectious agent was analyzed (Table 7). In keeping with current literature [23], mean PCT values were found to be higher in patients with gram negative as opposed to gram positive infection. However, the result did not demonstrate statistical significance (p = 0.7). The lack of statistical significant difference may be explained by the fact that extent of infection was not taken into consideration. Looking only at gram positive versus gram negative bacteremia may have yielded more meaningful results. Viral infections had a much smaller mean PCT value when compared to bacterial infections.

**Table 7:** Correlation between PCT value and infecting organism.

Variables	Total N = 192	Gram + N = 98	Gram - N = 90	Viral N = 4
Median PCT	2.5 (0.1–200.0)	1.7 (0.1–153.5)	3.8 (0.1–200.0)	3.5 (0.1–8.0)
Mean PCT ± SD	17.3 ± 34.1	13.7 ± 24.8	21.9 ± 42.2	3.7 ± 3.3

### Anatomical site of infection

PCT values were compared among five anatomical sites of infection using the Kruskal-Wallis test. At a significance level of 0.05, PCT values were found to be different among five anatomical sites. Procalcitonin values and their association with the site of infection were also analyzed (Table 8). Statistically significant differences were noted dependent on the anatomical site with sites typically infected by gram negative bacteria demonstrating higher procalcitonin values than those found in sites typically infected by gram positive bacteria. For instance, genitourinary and gastrointestinal infections tended to have higher procalcitonin values (17.4 ± 35.4 and 24.6 ± 36.8 respectively). In contrast, pneumonia had a mean procalcitonin of 10.0 ± 28.6, whereas musculoskeletal infection (cellulitis, osteomyelitis and septic arthritis) had procalcitonin values of 7.8 ± 14.3.

**Table 8:** Correlation between PCT value and anatomical site of infection.

Variables	Total N = 364	1 = *PNA N = 166	2 = *UTI N = 71	3 = *MSK N = 44	4 = *GI N = 27	5 = Other N = 56
Median PCT	1.6 (0.1–252.5)	1.4 (0.1–252.5)	2.5 (0.1–164.4)	1.3 (0.1–65.1)	5.1 (0.2–136.3)	2.7 (0.1–173.1)
Mean PCT ± SD	13.9 ± 31.6	10.0 ± 28.6	17.4 ± 35.4	7.8 ± 14.3	24.6 ± 36.8	20.4 ± 39.3

*PNA: Pneumonia, UTI: Urinary Tract infection, MSK: Musculoskeletal infection, GI: Gastrointestinal infection

### Chronic kidney disease

The results of this study revealed that patients with dialysis-dependent chronic kidney disease had statistically significant higher PCT values than those not on dialysis (Table 9).

**Table 9:** Correlation between PCT and dialysis.

Variables	Total N = 364	CKD with dialysis N = 16	CKD without dialysis N = 348
Median PCT	1.6 (0.1–252.5)	13.3 (0.1–200.0)	1.5 (0.1–252.5)
Mean PCT ± SD	13.9 ± 31.6	31.3 ± 51.5	13.1 ± 30.2

## Discussion

As noted above, PCT and SOFA score demonstrated a weak positive correlation. Other studies have also demonstrated an association between rising procalcitonin and SOFA scores [24, 25]and have noted a difference in PCT values in patients with sepsis versus septic shock [26, 27].In the analysis of bacteremia vs non-bacteremic infection, this study noted a statistically significant difference in PCT values between patients with bacteremia as demonstrated by positive blood cultures as opposed to patients with non-bacteremic infection and negative blood cultures, with the former having higher procalcitonin values than the latter. Similar results were found during an analysis of 280 patients with suspected bacteremia by Watanabe et al, where PCT concentrations were significantly higher in blood culture-positive cases (n = 55) than blood culture-negative cases (n = 235) (6.0±28.4 vs 0.29±0.5, respectively, *P* = 0.03) [28].Data analysis revealed that at a value of 3.05 ng/mL, the sensitivity and specificity of PCT for septic shock was found to be 63% and 65% respectively. Therefore, in the correct clinical context, PCT values approaching this threshold might prompt physicians to step up fluid and antibiotic management in order to prevent deterioration to septic shock.

The lack of a significant correlation between initial procalcitonin value and outcomes is disappointing but is in keeping with current literature which suggests that procalcitonin kinetics are a more useful marker of prognosis than one time measurements. A prospective study of patients admitted to the ICU determined that single serum PCT measurement, regardless of absolute value, has a discriminative impact but no prognostic significance during the first 2 days of therapy. PCT kinetics were found to be of prognostic value from day 3 of admission, and were found to be of earlier prognostic significance in comparison to changes in the patient's clinical condition evaluated by SOFA score kinetics [29].A meta-analysis of 21 studies with a total of 6007 patients concluded that although PCT may not be useful as a single index for assessing prognosis because of its moderate diagnostic accuracy, it may be useful when evaluated in combination with patients overall condition and other clinical indexes [30].

A study conducted in 2015 concluded that the 48-hour Δ SOFA score and the measurement of 24- and 48-hour PCT are useful prognostic markers in patients with sepsis and septic shock. The study recommended that a decrease in PCT clearance in the first 24 hours of treatment should prompt reassessment of the appropriateness and adequacy of treatment [31]. These findings imply that given the right clinical context and the absence of confounding factors, PCT measurements may be of value in the determination of level of care and may help to tailor treatment accordingly.

The negligible association between procalcitonin values and length of stay indicated that initial PCT values are not predictive of the duration of hospitalization. This particular analysis may have been confounded by the fact that patients with overwhelming infection may have died earlier, thereby resulting in a shorter length of stay. Length of stay can also be influenced by a number of other hospital events unrelated to infection. For instance, myocardial infarction, stroke, gastrointestinal bleeding and continued hospitalization while awaiting placement after discharge may have resulted in a protracted stay.

An unexpected finding was a lack of statistically significant difference in PCT value in patients with gram positive and gram negative infections. These findings are not supported by current literature [18]. A study which looked at 328 episodes of bacteremia found that serum PCT levels were significantly higher in patients with gram-negative sepsis than in those with gram-positive or fungal sepsis with an optimal cut-off value of 2.44 ng/mL in discriminating gram-negative sepsis from gram-positive sepsis. This yielded a sensitivity of 68.4% and a specificity of 77.1%[32]. A statistically significant difference in PCT value was noted when compared against the different anatomical sites of infection, which likely represents underlying etiological agents, given that pneumonia was associated with a lower PCT value than urinary tract and gastrointestinal infections, which are typically caused by gram negative organisms.

A statistically significant difference was noted with regards to PCT and dialysis dependent CKD. Patients who had sepsis and were on dialysis had higher a procalcitonin in comparison to patients who were not on dialysis. This is consistent with current literature [22], which suggests that procalcitonin values tend to be higher on average in patients undergoing dialysis during periods of illness as well as health. This phenomenon is thought to be likely secondary to the pro-inflammatory state that results in patients with chronic kidney disease necessitating dialysis [22]. A study of 62 patients on maintenance hemodialysis found that procalcitonin concentrations were elevated in 57% of patients and showed a mean PCT of 0.69±0.81 ng/ml, which was slightly above the upper limit of normal (0.5 ng/ml) even though only 18% of the total population studied had a current bacterial infection [21]. Procalcitonin levels were significantly higher in the group with infection than those without (1.15±1.5 vs 0.58±0.38, P = 0.03). These findings suggest that for patients on dialysis, it may be appropriate to establish of a higher threshold of normal for PCT in comparison to the general population [21].

## Conclusion

Procalcitonin has proven itself to be a reliable marker of infection, with a higher specificity for bacterial infections than other acute phase reactants. However, although initial procalcitonin value may be helpful in the determination of illness severity, it may not always be a reliable prognostic indicator and oftentimes carries little significance as a standalone value. It is important to keep in mind that procalcitonin values may be influenced by preexisting comorbid conditions such as chronic kidney disease and congestive heart failure, which have been shown in studies to be associated with higher PCT values at baseline. Indeed, procalcitonin can provide invaluable information when viewed as one piece of a clinical puzzle, and must always be interpreted in the clinical context. Therefore, it is most powerful when the interpreting physician is aware of how values are influenced by the different clinical scenarios presented in this article.

## Limitations

Given that this was a retrospective chart review, the time of procalcitonin measurement from initial presentation could not be standardized, but tended to fall within the first 24 hours of presentation. Even though this study sought to determine the nature of the relationship between initial procalcitonin value and initial SOFA score, an evolving clinical course or the occurrence of unexpected events (nosocomial infection, change in status from full treatment to comfort measures etc.) may have served to confound the results obtained. Additionally, this study took into consideration only the initial procalcitonin value. Serial values were not measured. As noted above, a change in PCT as opposed to a single value may be more predictive of outcome.

As mentioned in the methodology section, an arterial blood gas analysis was unavailable for a minority of the patients, thus leading to an impediment when calculating the SOFA score. Even though clinical calculators such as the SOFA score seek to minimize or altogether eliminate subjectivity and operator-dependent differences, it is still possible that methods of data gathering used by the two researchers were different enough to have yielded incongruent scores. The effect of this was minimized by the implementation of a standardization process for data gathering.

## Supporting information

S1 Table Data collection sheet. (XLSX)

S2 Table Interpretation key. (XLSX)

S1 Fig Correlation between SOFA score and p-value. (RTF)
