# Supplementary material for: The use of procalcitonin in the determination of severity of sepsis, patient outcomes and infection characteristics
Source: PLoS One. 2018 Nov 14;13(11):e0206527. doi: 10.1371/journal.pone.0206527 (PMC6235293; doi:10.1371/journal.pone.0206527)
Supplement: S1 Fig — (RTF) [file pone.0206527.s003.rtf]

The SAS System

The CORR Procedure	

3 With Variables:	SOFA_score        LOS               Age	
1      Variables:	Initial_PCT_value	


Simple Statistics	
Variable	N	Mean	Std Dev	Median	Minimum	Maximum	Label	
SOFA_score	364	5.06044	3.49524	4.00000	0	23.00000	SOFA score	
LOS	364	8.61538	6.22847	7.00000	1.00000	48.00000	LOS	
Age	364	61.31593	12.63349	63.00000	20.00000	86.00000	Age	
Initial_PCT_value	364	13.87909	31.57429	1.64500	0.05000	252.50000	Initial PCT value	

Spearman Correlation Coefficients, N = 364
Prob > |r| under H0: Rho=0	
	Initial_PCT_value	
SOFA_score
SOFA score	0.32932
<.0001	
LOS
LOS	0.17207
0.0010	
Age
Age	0.01332
0.8000	


The SAS System

The CORR Procedure	

1 With Variables:	LOS	
1      Variables:	Initial_PCT_value	


Simple Statistics	
Variable	N	Mean	Std Dev	Median	Minimum	Maximum	Label	
LOS	316	8.36709	5.88067	7.00000	1.00000	48.00000	LOS	
Initial_PCT_value	316	13.03443	30.50421	1.52500	0.05000	252.50000	Initial PCT value	


Spearman Correlation Coefficients, N = 316
Prob > |r| under H0: Rho=0	
	Initial_PCT_value	
LOS
LOS	0.20551
0.0002	


The SAS System

The CORR Procedure	

1 With Variables:	LOS	
1      Variables:	Initial_PCT_value	


Simple Statistics	
Variable	N	Mean	Std Dev	Median	Minimum	Maximum	Label	
LOS	48	10.25000	8.04376	10.00000	1.00000	35.00000	LOS	
Initial_PCT_value	48	19.43979	37.77546	1.87000	0.05000	173.10000	Initial PCT value	


Spearman Correlation Coefficients, N = 48
Prob > |r| under H0: Rho=0	
	Initial_PCT_value	
LOS
LOS	-0.05493
0.7108	
